# Supplementary material for: BRD2 upregulation as a pan-cancer adaptive resistance mechanism to BET inhibition
Source: Cell Mol Biol Lett. 2026 May 2;31:106. doi: 10.1186/s11658-026-00922-y (PMC13362212; doi:10.1186/s11658-026-00922-y)

Supplementary Fig. 1 **BRD2 upregulation by JQ1 across additional cancer types.**

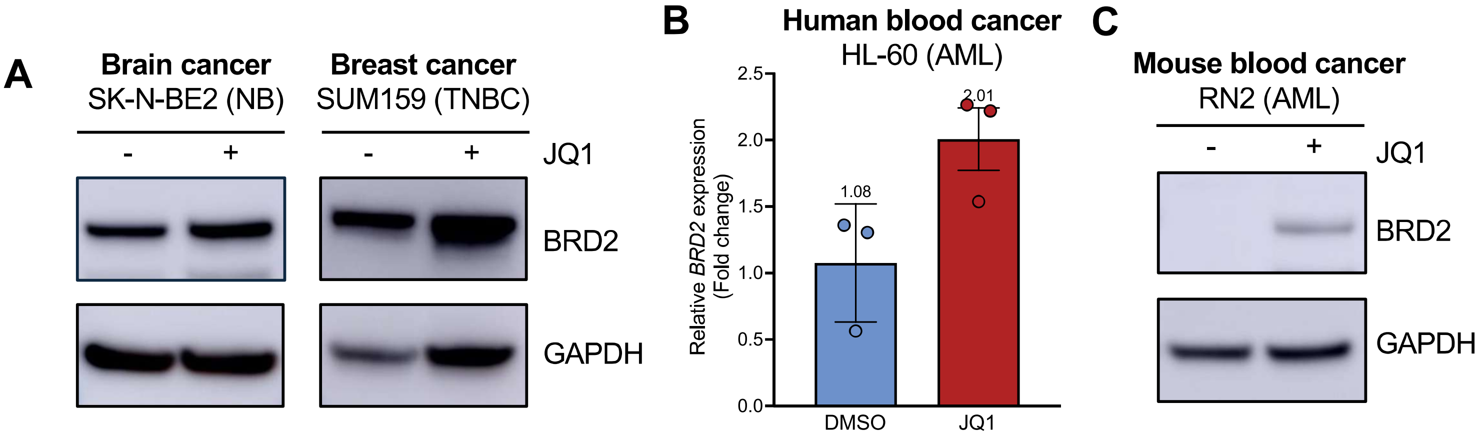

Supplementary Fig. 2

BRD2 induction by BET inhibition but not by BRD4 KD.

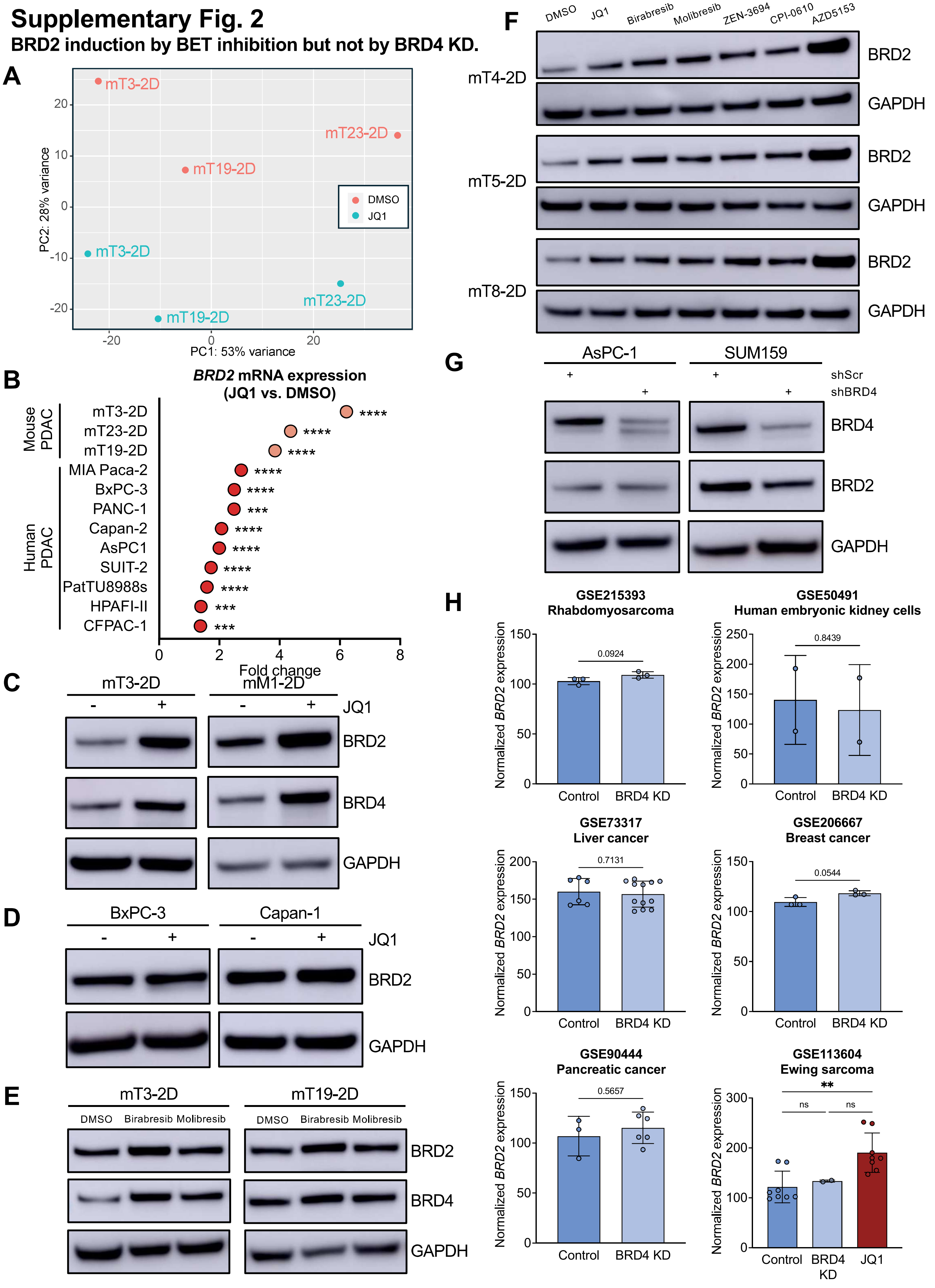

Supplementary Fig. 3 NFYA emerges as a candidate regulator of BRD2.

A

	Gene	Function	hsa-u.4 / BRD2	hsa-r.6 / BRD2	hsa-m.7 / BRD2	hsa-m2.4 / BRD2
1	PPP1R10	protein phosphatase 1 regulatory subunit 10	8.9	8.1	5	7.7
2	NXF1	nuclear RNA export factor 1	8	6.9	2.9	7.1
3	POLR2A	RNA polymerase II subunit A	7.6	6.7	2.8	6.6
4	RBM22	RNA binding motif protein 22	7.5	7.8	2.6	5.3
5	FAM193A	family with sequence similarity 193 member A	7.4	7.4	1.1	5.6
6	BRPF3	bromodomain and PHD finger containing 3	7.1	5.5	1.7	6.9
7	PRRC2A	proline rich coiled-coil 2A	6.8	6.2	3.4	5.8
8	BTN2A1	butyrophilin subfamily 2 member A1	6.8	6.7	1.9	5.1
9	TRIM39	tripartite motif containing 39	6.7	5.9	2.8	5.9
10	SRCAP	Snf2 related CREBBP activator protein	6.6	5	1.8	6.6
11	BRPF1	bromodomain and PHD finger containing 1	6.6	6.1	1.9	5.4
12	RSRC2	arginine and serine rich coiled-coil 2	6.6	7.1	2.9	4.4
13	KDM2A	lysine demethylase 2A	6.5	6.5	2.7	4.9
14	SCAF4	SR-related CTD associated factor 4	6.2	5.8	1	5.1
15	FAM53C	family with sequence similarity 53 member C	6.2	5.4	2.1	5.6
16	CCNL1	cyclin L1	6.2	6.8	1.3	4.1
17	EP300	E1A binding protein p300	6.2	5.4	1.9	5.5
18	ABCF1	ATP binding cassette subfamily F member 1	6.2	6	2.2	4.8
19	PHF12	PHD finger protein 12	6.1	5.8	1.2	5
20	CNOT4	CCR4-NOT transcription complex subunit 4	6.1	7.5	1.5	3.2
21	TRIM26	tripartite motif containing 26	6.1	7	2.6	3.7
22	ZNF592	zinc finger protein 592	6	4.1	0.6	6.4
23	ATXN2L	ataxin 2 like	5.9	5.1	1.9	5.3
24	ZNF335	zinc finger protein 335	5.8	5.3	1.8	5
25	SETD5	SET domain containing 5	5.8	6	2.3	4.2
26	CDK12	cyclin dependent kinase 12	5.8	6	0.6	4.2
27	NUP153	nucleoporin 153	5.8	5.6	1.8	4.5
28	CREBBP	CREB binding protein	5.8	5.2	0.9	5
29	SRRM2	serine/arginine repetitive matrix 2	5.8	4	5.1	6.2
30	**NFYA**	**nuclear transcription factor Y subunit alpha**	**5.7**	**5.6**	**1.7**	**4.4**

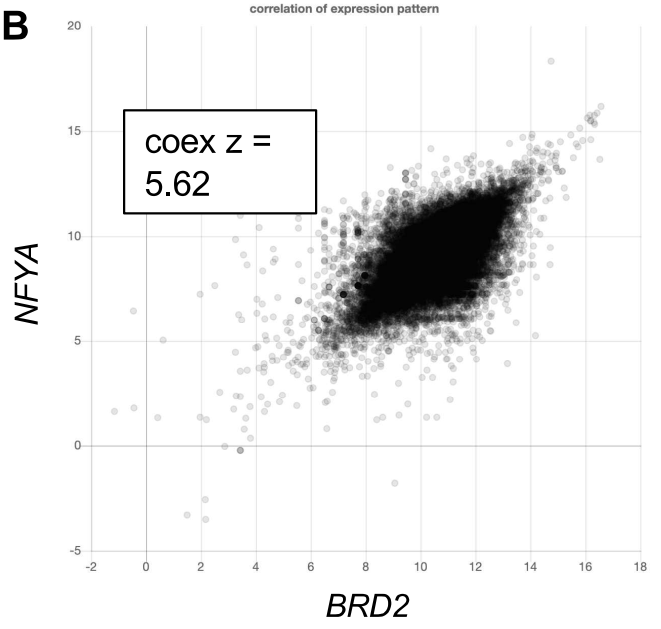

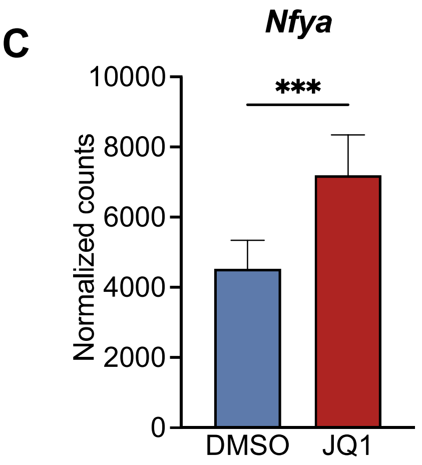

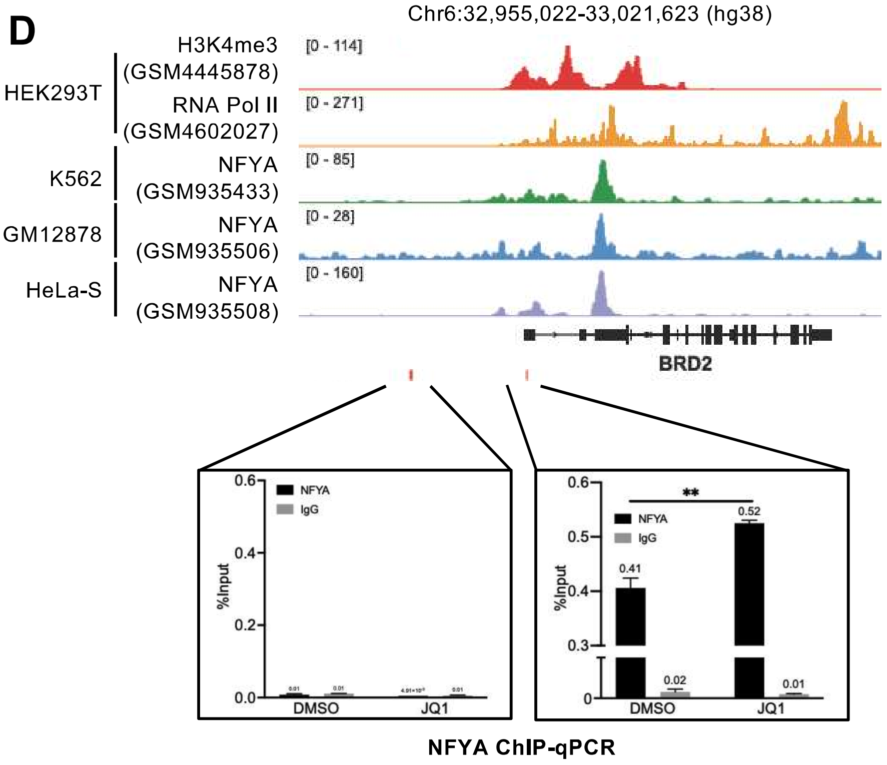

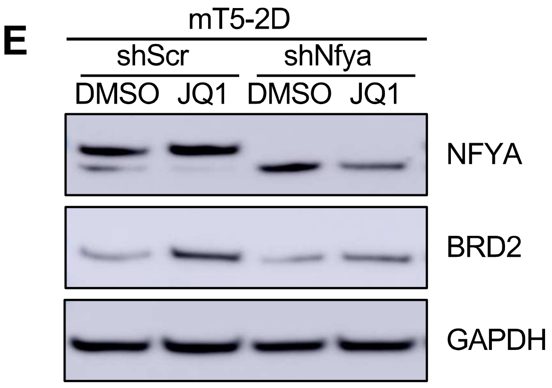

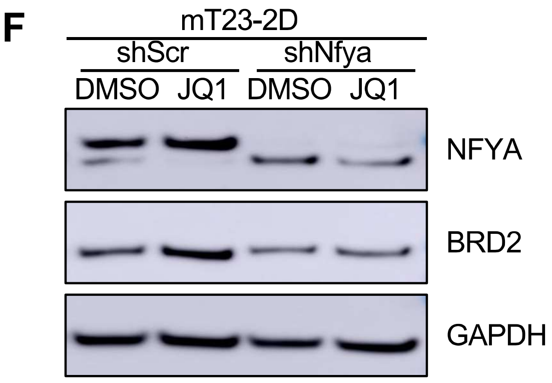

Supplementary Fig. 4 **BRD2 KD enhances sensitivity to BET inhibition across murine PDAC models.**

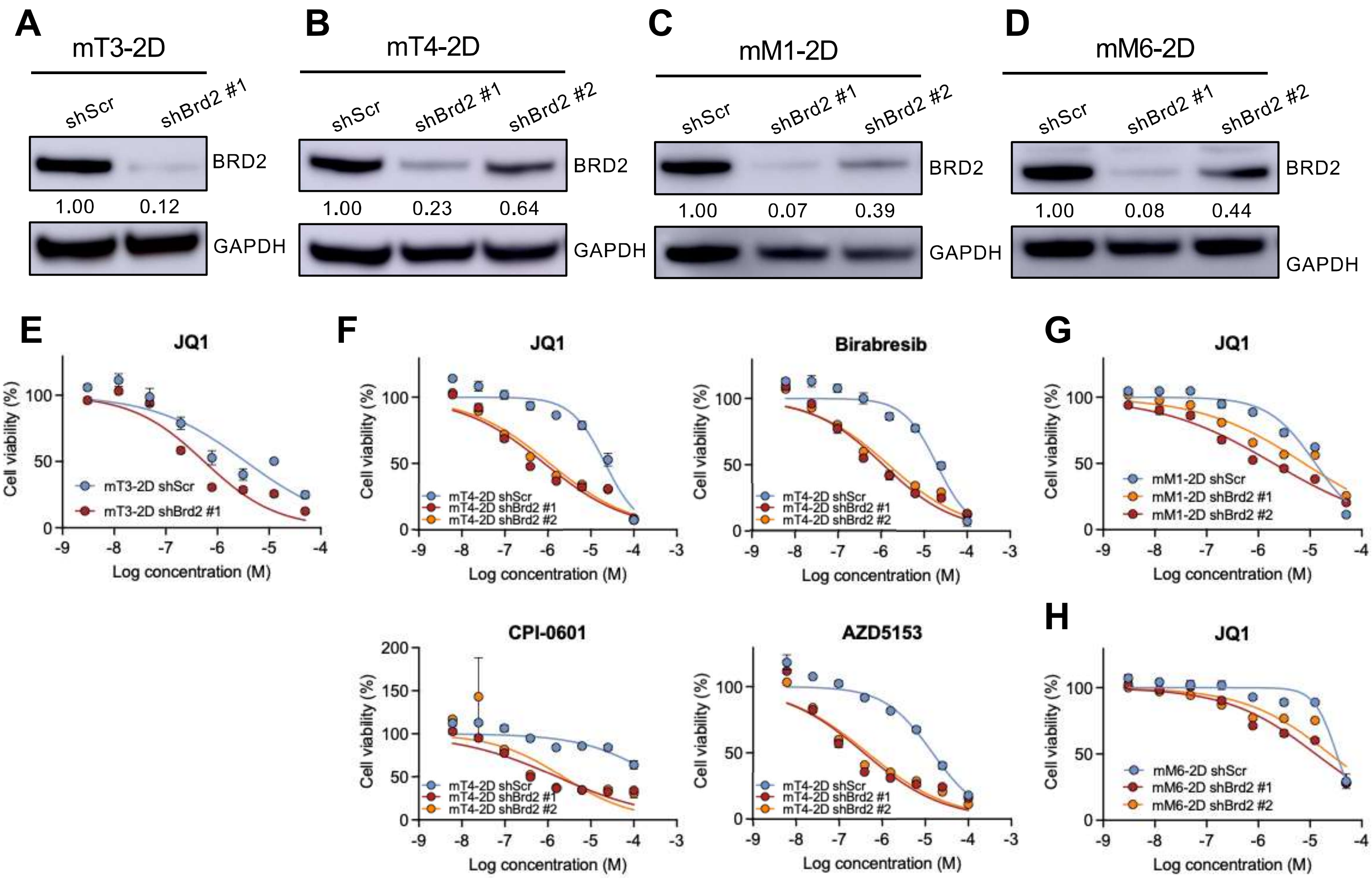

Supplementary Fig. 4 BRD2 KD enhances sensitivity to BET inhibition across murine PDAC models.

I

$LogIC_{50}$ and $\Delta LogIC_{50}$ of BETi sensitivity after BRD2 KD across cell lines

Drugs	Cell lines		$LogIC_{50}$ (M)	95% CI	$\Delta LogIC_{50}$
JQ1	mT23-2D	shScr	-4.556	-4.733 to -4.310	
		shBrd2 #1	-5.634	-5.760 to -5.506	1.078
	mT3-2D	shScr	-5.455	-5.769 to -5.104	
		shBrd2 #1	-6.258	-6.464 to -6.040	0.803
	mT4-2D	shScr	-4.667	-4.805 to -4.534	
		shBrd2 #1	-6.081	-6.291 to -5.864	1.414
		shBrd2 #2	-5.956	-6.128 to -5.782	1.289
	mM1-2D	shScr	-4.896	-5.018 to -4.776	
		shBrd2 #1	-5.704	-5.828 to -5.576	0.808
		shBrd2 #2	-5.112	-5.287 to -4.920	0.216
	mM6-2D	shScr	-4.486	-4.565 to -4.410	
		shBrd2 #1	-4.891	-5.047 to -4.718	0.405
		shBrd2 #2	-4.618	-4.844 to -4.305	0.132
	A549	shScr	-4.092	NE	
		shBRD2 #1	-5.105	-5.317 to -4.887	1.013
	U251	shScr	-3.979	NE	
		shBRD2 #1	-5.305	-5.576 to -5.022	1.326
	U2OS	shScr	-4.382	-4.538 to -4.249	
		shBRD2 #1	-6.163	-6.493 to -5.842	1.781
	Capan-2	shScr	-3.13	-3.314 to -2.871	
		shBRD2 #1	-4.845	-5.080 to -4.612	1.715
	CFPAC-1	shScr	-4.367	-4.514 to -4.233	
		shBRD2 #1	-4.839	-4.528 to -4.229	0.472
	BxPC-3	shScr	-5.386	-5.603 to -5.174	
		shBRD2 #1	-6.425	-6.638 to -6.224	1.039
Birabresib	mT23-2D	shScr	-4.551	-4.744 to -4.362	
		shBrd2 #1	-5.127	-5.316 to -4.933	0.576
	mT4-2D	shScr	-4.701	-4.845 to -4.561	
		shBrd2 #1	-5.949	-6.135 to -5.758	1.248
		shBrd2 #2	-5.825	-5.995 to -5.651	1.124
Molibresib	mT23-2D	shScr	-3.792	-4.045 to -3.236	
		shBrd2 #1	-4.311	-4.546 to -4.013	0.519
ZEN-3694	mT23-2D	shScr	-4.104	-4.401 to -3.686	
		shBrd2 #1	-4.52	-4.742 to -4.271	0.416
CPI-0610	mT23-2D	shScr	-4.003	-4.326 to -3.412	
		shBrd2 #1	-4.966	-5.265 to -4.619	0.963
	mT4-2D	shScr	-3.467	-3.872 to -2.656	
		shBrd2 #1	-5.651	-6.416 to -4.673	2.184
		shBrd2 #2	-5.787	-6.104 to -5.438	2.32
AZD5153	mT23-2D	shScr	-4.481	-4.708 to -4.229	
		shBrd2 #1	-5.497	-5.732 to -5.250	1.016
	mT4-2D	shScr	-4.832	-4.983 to -4.678	
		shBrd2 #1	-6.425	-6.709 to -6.124	1.593
		shBrd2 #2	-6.342	-6.539 to -6.141	1.51

IC_{50}: Half maximal inhibitory concentration
CI: Confidence interval
NE: Not estimable

Supplementary Fig. 4 **BRD2 KD enhances sensitivity to BET inhibition across murine PDAC models.**

J

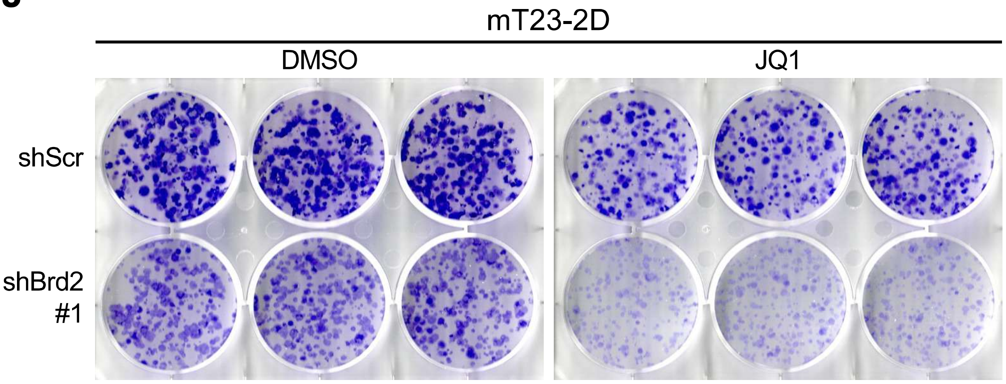

M

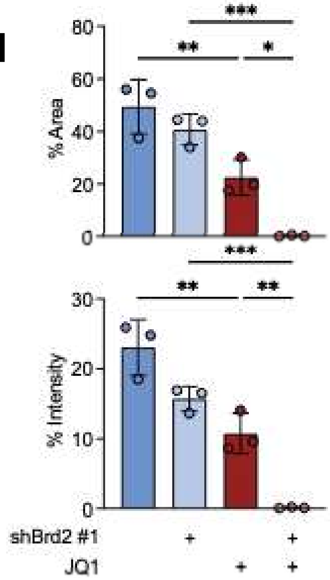

K

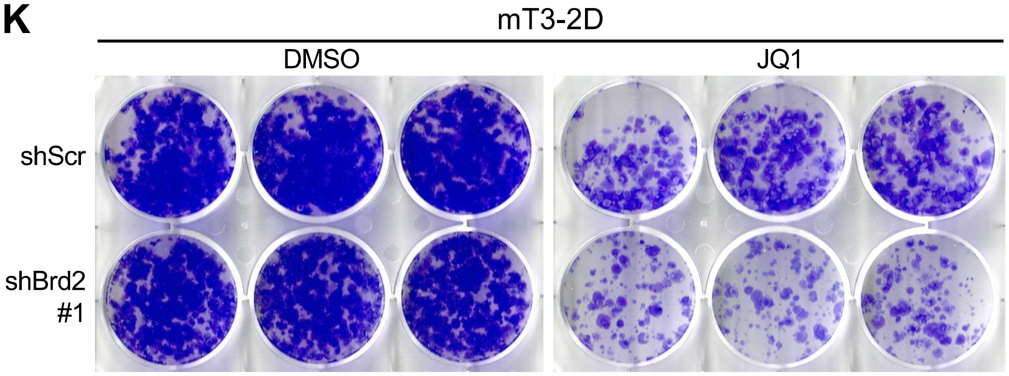

N

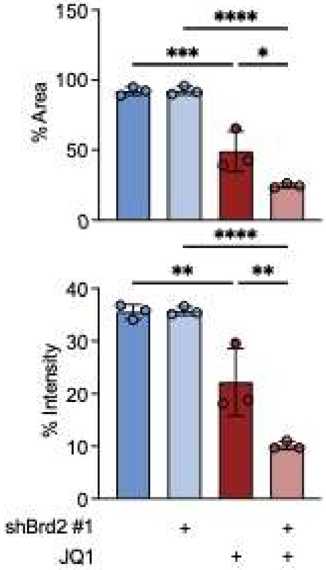

L

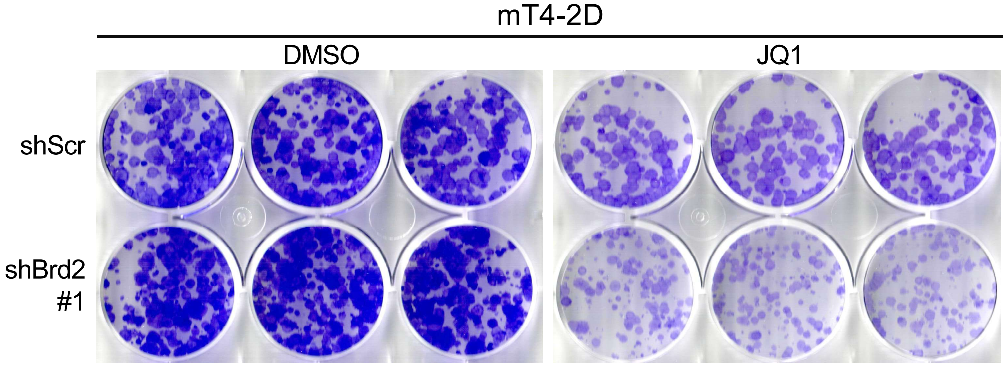

O

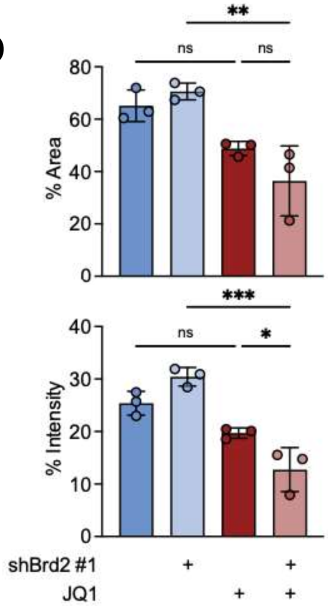

Supplementary Fig. 5 **Body weight of mice was maintained during JQ1 treatment.**

A

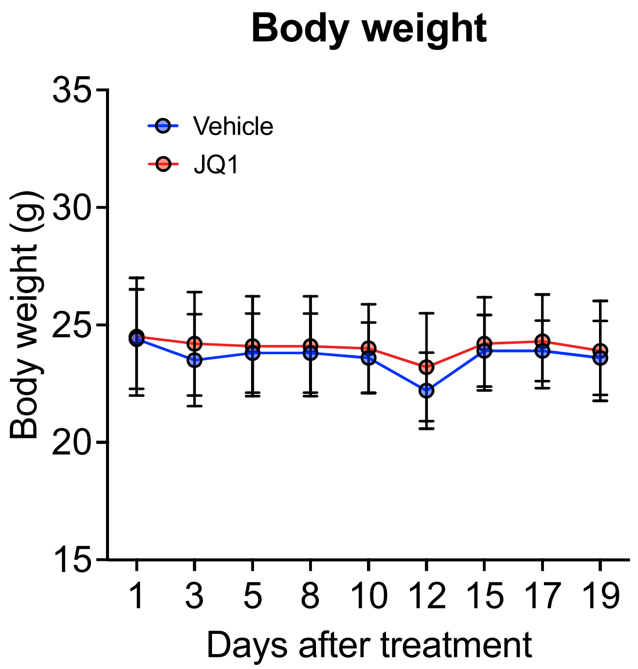

Supplementary Fig. 6 Basal BRD4/BRD2 levels and chromatin occupancy do not predict JQ1 response.

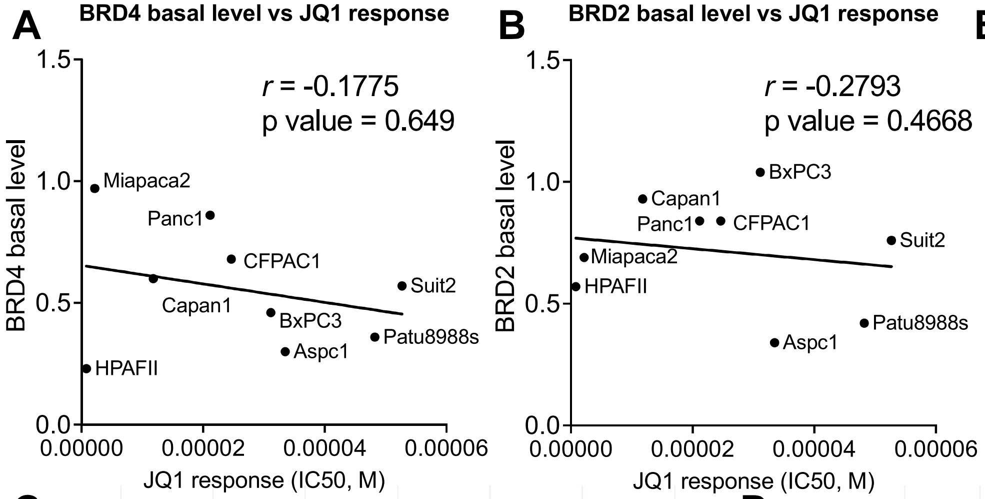

E

Drugs	Cell lines	$LogIC_{50}$ (M)	95% CI
JQ1	mT3-2D	-6.989	-7.139 to -6.841
	mT23-2D	-4.583	-4.775 to -4.419
Molibresib	mT3-2D	-5.875	-5.969 to -5.779
	mT23-2D	-3.605	-3.754 to -3.393
Birabresib	mT3-2D	-6.598	-6.713 to -6.479
	mT23-2D	-4.623	-4.751 to -4.496

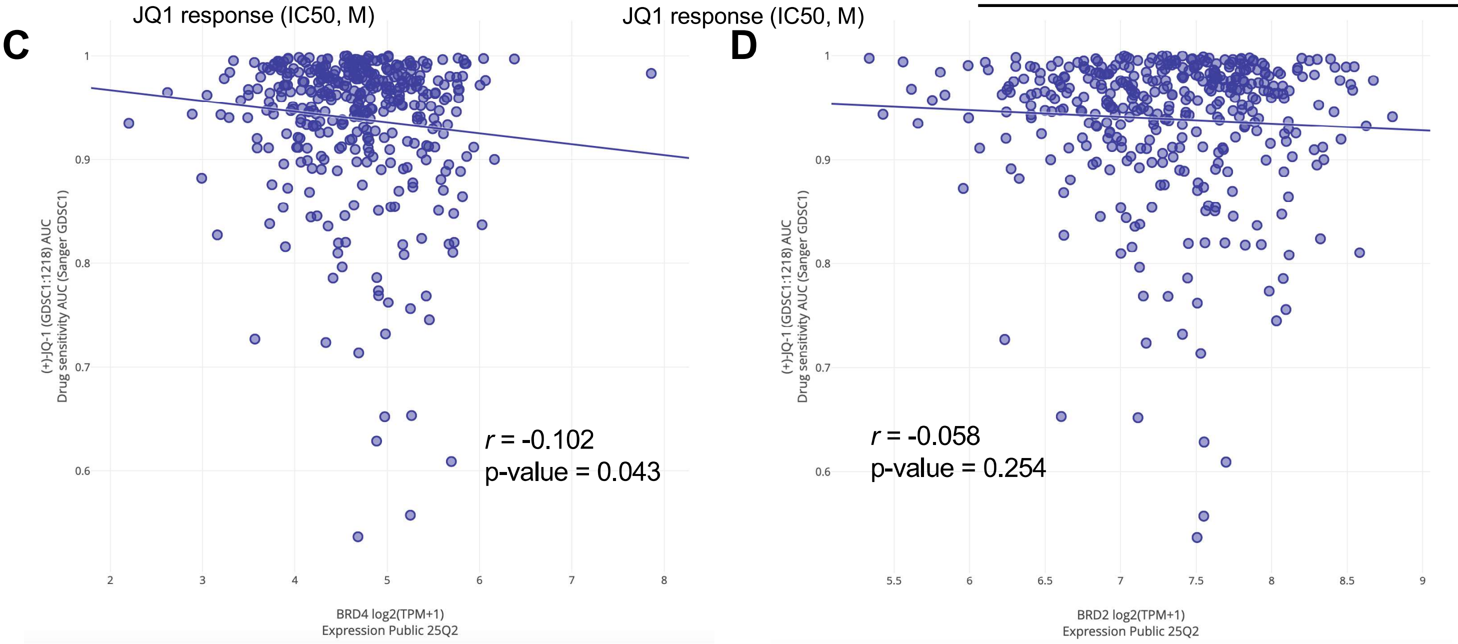

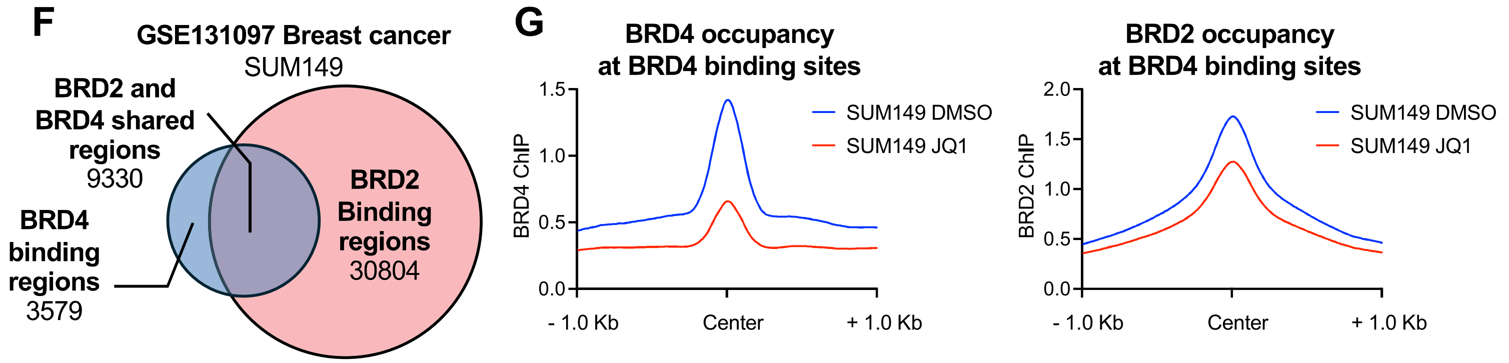

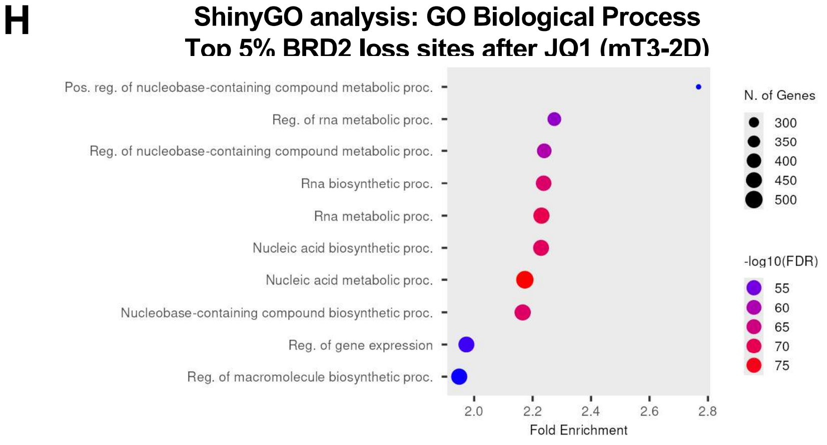

Supplement: Supplementary file 1 — Supplementary Material 1. Supplementary Fig. 1 BRD2 upregulation by JQ1 across additional cancer types. (A) Western blot analysis of BRD2 in brain and breast cancer cells treated with DMSO or JQ1. (B–C) RT-qPCR and immunoblot analysis of BRD2 expression in human and mouse blood cancer cells, showing increased BRD2 levels upon JQ1 treatment (1 µM, 24 hours). Supplementary Fig. 2 BRD2 induction by BET inhibition but not by BRD4 KD. (A) PCA of RNA-seq profiles from three different murine PDAC cell lines treated with DMSO or JQ1, showing distinct clustering of JQ1-treated samples. (B) RT-qPCR analysis of BRD2 expression in multiple murine and human PDAC cell lines treated with JQ1, revealing significant induction across models. Statistical significance was determined using a two-tailed Student’s t-test (***p < 0.001, ****p < 0.0001) (C–D) Western blot analysis of BRD2 and BRD4 expression in murine (mT3-2D, mM1-2D) and human (BxPC-3, Capan-1) PDAC cells after JQ1 treatment. (E) Immunoblot analysis of BRD2 and BRD4 expression following treatment with JQ1, Birabresib or Molibresib in mouse PDAC cells (mT3-2D and mT19-2D). (F) Western blot analysis of BRD2 expression in murine PDAC cell lines (mT4-2D, mT5-2D, and mT8-2D) treated with different BETi (JQ1, Birabresib, Molibresib, ZEN-3694, CPI-0610, AZD5153), showing consistent BRD2 upregulation across inhibitors compared to control. (G) Western blot analysis of BRD2 expression after shRNA-mediated BRD4 KD in PDAC AsPC-1 and breast cancer SUM159 cells, showing that BRD4 depletion did not result in BRD2 induction by JQ1. (H) Analysis of publicly available RNA-seq datasets (GEO) across multiple cancer types, showing that BRD4 KD generally did not increase BRD2 expression, whereas JQ1 significantly induced BRD2. Statistical significance was determined by two-tailed Student’s t-test (**p < 0.01). Supplementary Fig. 3 NFYA emerges as a candidate regulator of BRD2. (A) List of top 30 BRD2-interacting transcriptional regulators iden [file 11658_2026_922_MOESM1_ESM.pdf]
