# Supplementary material for: BRD2 upregulation as a pan-cancer adaptive resistance mechanism to BET inhibition
Source: Cell Mol Biol Lett. 2026 May 2;31:106. doi: 10.1186/s11658-026-00922-y (PMC13362212; doi:10.1186/s11658-026-00922-y)
Supplement: Supplementary file 2 — Supplementary Material 2. Table S1. Cell lines and culture conditions used in this study. Table S2. BET inhibitors used in this study. Table S3. shRNA constructs used in this study.Table S4. Antibodies used in this study. Table S5. List of primers and sequences for RT-qPCR and ChIP-qPCR. Table S6. Publicly available RNA-seq datasets used for BRD2 expression analysis. Table S7. Publicly available GEO RNA-seq datasets used to analyze BRD2 expression under BRD4 knockdown and BET inhibition. Table S8. Publicly available BRD2 and BRD4 ChIP-seq dataset used in this study. Table S9. ChIP-seq datasets for NFYA, H3K4me3, RNA Pol II occupancy at the BRD2 locus. [file 11658_2026_922_MOESM2_ESM.docx]

**Table S1.** Cell lines and culture conditions used in this study

| KPC-derived mouse pancreatic ductal adenocarcinoma (PDAC) cell lines | | | | | | |
| --- | --- | --- | --- | --- | --- | --- |
| Cell line | **Type** | **Origin** | **Culture medium** | **Source** | | **Catalog no.** |
| mT3-2D | PDAC | Pancreas | DMEM + 10% FBS + 1% P/S | Gift from Dr. David Tuveson Cold Spring Harbor Laboratory | | |
| mT19-2D | PDAC | Pancreas | DMEM + 10% FBS + 1% P/S |  |  |  |
| mT23-2D | PDAC | Pancreas | DMEM + 10% FBS + 1% P/S |  |  |  |
| mT4-2D | PDAC | Pancreas | DMEM + 10% FBS + 1% P/S |  |  |  |
| mT5-2D | PDAC | Pancreas | DMEM + 10% FBS + 1% P/S |  |  |  |
| mT8-2D | PDAC | Pancreas | DMEM + 10% FBS + 1% P/S |  |  |  |
| mM1-2D | PDAC | Pancreas | DMEM + 10% FBS + 1% P/S |  |  |  |
| mM6-2D | PDAC | Pancreas | DMEM + 10% FBS + 1% P/S |  |  |  |
| Other mouse cell lines | | | | | | |
| Cell line | **Type** | **Origin** | **Culture medium** | **Source** | | **Catalog no.** |
| RN2 | AML | Bone marrow | DMEM + 10% FBS + 1% P/S | Gift from Dr. Christopher R. Vakoc Cold Spring Harbor Laboratory | | |
| HEK293T | HEK | Kidney | DMEM + 10% FBS + 1% P/S | ATCC | CRL-3216 | |
| Human PDAC cell lines | | | | | | |
| Cell line | **Type** | **Origin** | **Culture medium** | **Source** | | **Catalog no.** |
| AsPC-1 | PDAC | Pancreas | RPMI-1640 + 10% FBS + 1% P/S | ATCC | | CRL-1682 |
| BxPC-3 | PDAC | Pancreas | RPMI-1640 + 10% FBS + 1% P/S | ATCC | | CRL-1687 |
| CFPAC-1 | PDAC | Pancreas | RPMI-1640 + 10% FBS + 1% P/S | ATCC | | CRL-1918 |
| MIA PaCa-2 | PDAC | Pancreas | RPMI-1640 + 10% FBS + 1% P/S | ATCC | | CRL-1420 |
| PANC-1 | PDAC | Pancreas | RPMI-1640 + 10% FBS + 1% P/S | ATCC | | CRL-1469 |
| PaTu8988s | PDAC | Pancreas | RPMI-1640 + 10% FBS + 1% P/S | Creative Bioarray | | CSC-C0326 |
| SUIT-2 | PDAC | Pancreas | RPMI-1640 + 10% FBS + 1% P/S | Creative Bioarray | | CSC-C6655J |
| Capan-1 | PDAC | Pancreas | RPMI-1640 + 20% FBS + 1% P/S | ATCC | | HTB-79 |
| Capan-2 | PDAC | Pancreas | McCoy’s 5A + 10% FBS + 1% P/S | ATCC | | HTB-80 |
| HPAF-II | PDAC | Pancreas | EMEM + 10% FBS + 1% P/S | ATCC | | CRL-1997 |

| Other human cell lines | | | |  |  |
| --- | --- | --- | --- | --- | --- |
| Cell line | **Type** | **Origin** | **Culture medium** | **Source** | **Catalog no.** |
| A549 | NSCLC | Lung | DMEM + 10% FBS + 1% P/S | ATCC | CCL-185 |
| Huh7 | HCC | Liver | DMEM + 10% FBS + 1% P/S | Cytion | 300156 |
| PC3 | CRPC | Prostate | DMEM + 10% FBS + 1% P/S | ATCC | CRL-1435 |
| SK-N-BE2 | NB | Brain | DMEM + 10% FBS + 1% P/S | ATCC | CRL-2271 |
| U251 | GB | Brain | DMEM + 10% FBS + 1% P/S | Sigma-Aldrich | 09063001 |
| U2OS | OS | Bone | McCoy’s 5A + 10% FBS + 1% P/S | ATCC | HTB-96 |
| HL-60 | AML | Bone marrow | RPMI-1640 + 10% FBS + 1% P/S | ATCC | CCL-240 |
| SUM159 | TNBC | Breast | Advanced DMEM/F-12 + 5% FBS + 10mM HEPES + 1µg/ml hydrocortisone + 5µg/ml insulin | Cytion | 305116 |
| MDA-MB-231 | TNBC | Breast | Advanced DMEM/F-12 + 5% FBS + 10mM HEPES + 1µg/ml hydrocortisone + 5µg/ml insulin | ATCC | HTB-26 |

**Abbreviations:** AML, acute myeloid leukemia; ATCC, American Type Culture Collection; CRPC, castration-resistant prostate cancer; DMEM, Dulbecco’s modified Eagle medium; FBS, fetal bovine serum; GB, glioblastoma; HCC, hepatocellular carcinoma; HEPES, 4-(2-hydroxyethyl)-1-piperazineethanesulfonic acid; NB, neuroblastoma; NSCLC, non–small cell lung cancer; OS, osteosarcoma; P/S, penicillin–streptomycin; RPMI, Roswell Park Memorial Institute medium; TNBC, triple-negative breast cancer.

**Table S2.** BET inhibitors used in this study

| Compound Name | Source | Catalog Number |
| --- | --- | --- |
| JQ1 | MedChemExpress | HY-13030 |
| Molibresib (I-BET762) | MedChemExpress | HY-13032 |
| Birabresib (OTX-015) | MedChemExpress | HY-15743 |
| AZD5153 | MedChemExpress | HY-100653 |
| CPI-203 | MedChemExpress | HY-15846 |
| ZEN-3694 | Selleckchem | E1517 |

**Table S3.** shRNA constructs used in this study

| Non-targeting shRNA control construct | |
| --- | --- |
| shRNA construct | **Sequence (5’ 🡪 3’)** |
| shScramble (shScr) | Forward: CCGGCCTAAGGTTAAGTCGCCCTCGCTC  GAGCGAGGGCGACTTAACCTTAGGTTTTTG |
|  | Reverse:  AATTCAAAAACCTAAGGTTAAGTCGCCCT  CGCTCGAGCGAGGGCGACTTAACCTTAGG |

| Mouse shRNA construct | | |
| --- | --- | --- |
| shRNA construct | **Target ID & sequence** | **Sequence (5’ 🡪 3’)** |
| shBrd2  #1 | TRCN0000362395 (CCCTCTCTACGTGATTCAAAT) | Forward: CCGGCCCTCTCTACGTGATTCAAATCTCG AGATTTGAATCACGTAGAGAGGGTTTTTG |
|  |  | Reverse:  AATTCAAAAACCCTCTCTACGTGATTCAA ATCTCGAGATTTGAATCACGTAGAGAGGG |
| shBrd2  #2 | TRCN0000362394  (CCCGGAAGCCCTACACTATTA) | Forward:  CCGGCCCGGAAGCCCTACACTATTACTCG  AGTAATAGTGTAGGGCTTCCGGGTTTTTG |
|  |  | Reverse:  AATTCAAAAACCCGGAAGCCCTACACTAT  TACTCGAGTAATAGTGTAGGGCTTCCGGG |
| shBrd4 | TRCN0000088480 (GCGGCAGCTAAGTCTAGATAT) | Forward: CCGGGCGGCAGCTAAGTCTAGATATCTC GAGATATCTAGACTTAGCTGCCGCTTTTTG |
|  |  | Reverse:  AATTCAAAAAGCGGCAGCTAAGTCTAGAT ATCTCGAGATATCTAGACTTAGCTGCCGC |
| shNfya | TRCN0000084442  (CGCATCCTTAAGAGGAGACAA)  **Bacterial glycerol stock from Millipore Sigma** | Forward: CCGGCGCATCCTTAAGAGGAGACAACTC  GAGTTGTCTCCTCTTAAGGATGCGTTTTTG |
|  |  | Reverse:  AATTCAAAAACGCATCCTTAAGAGGAGAC  AACTCGAGTTGTCTCCTCTTAAGGATGCG |
| Human shRNA construct | | |
| shRNA construct | **Target sequence ID** | **Sequence (5’ 🡪 3’)** |
| shBRD2  #1 | TRCN0000006310 (CCCTGCCTACAGGTTATGATT) | Forward:  CCGGCCCTGCCTACAGGTTATGATTCTCG  AGAATCATAACCTGTAGGCAGGGTTTTTG |
|  |  | Reverse:  AATTCAAAAACCCTGCCTACAGGTTATGA  TTCTCGAGAATCATAACCTGTAGGCAGGG |
| shBRD2  #2 | TRCN0000006311  (CCTATGGACATGGGTACTATT) | Forward:  CCGGCCTATGGACATGGGTACTATTCTCG  AGAATAGTACCCATGTCCATAGGTTTTTG |
|  |  | Reverse:  AATTCAAAAACCTATGGACATGGGTACTA  TTCTCGAGAATAGTACCCATGTCCATAGG |
| shBRD4 | TRCN0000021427 (CCTGGAGATGACATAGTCTTA) | Forward:  CCGGCCTGGAGATGACATAGTCTTACTC  GAGTAAGACTATGTCATCTCCAGGTTTTTG |
|  |  | Reverse:  AATTCAAAAACCTGGAGATGACATAGTCT  TACTCGAGTAAGACTATGTCATCTCCAGG |

**Table S4.** Antibodies used in this study

| Antibody | Source | Catalog Number | Dilution |
| --- | --- | --- | --- |
| BRD2 | Cell Signaling Technology | 5848S | 1:1000 |
| BRD4 | Novus Biologicals | NBP2-76393 | 1:1000 |
| NFYA | Fortis Life Sciences | A302-105A-T | 1:5000 |
| GAPDH | ProSci | 3783 | 1:1000 |
| Vinculin | Cell Signaling Technology | 13901 | 1:1000 |
| Donkey anti-rabbit IgG (H+L) HRP | Invitrogen | A16023 | 1:5000 |

**Table S5.** List of primers and sequences for RT-qPCR and ChIP-qPCR

| RT-qPCR |  |  |
| --- | --- | --- |
| Primers | Direction | Sequence (5′ 🡪 3′) |
| *Brd2* | Forward | ATGCTGCAAAACGTGACTCC |
|  | Reverse | AAGCTGGTACAGAAGCCATTG |
| *Brd4* | Forward | AGTGTCTTTGACCCTATTAGCCA |
|  | Reverse | CATGCTGGTTGAGATGGGG |
| *Gapdh* | Forward | TTCACCACCATGGAGAAGGC |
|  | Reverse | CCCTTTTGGCTCCACCCT |
| *BRD2* | Forward | CGGCTTATGTTCTCCAACTGCTA |
|  | Reverse | GGCAGTAGAGACTGGTAAAGGC |
| *BRD4* | Forward | ACCTCCAACCCTAACAAGCC |
|  | Reverse | TTTCCATAGTGTCTTGAGCACC |
| *GAPDH* | Forward | CCAAGGAGTAAGACCCCTGG |
|  | Reverse | AGGGGAGATTCAGTGTGGTG |
| ChIP-qPCR |  |  |
| *BRD2* | Forward | TTATAGCGCTCTGGGTTTCGG |
|  | Reverse | GACCAGCTTTCCGAACGTTC |
| Intergenic | Forward | ACCAGGTTGACCTTGGGTAAC |
|  | Reverse | TTTGGCAGTTTGGTGAAGCC |

**Table S6.** Publicly available RNA-seq datasets used for *BRD2* expression analysis

| Prostate cancer | | |
| --- | --- | --- |
| GEO accession | **Cancer type** | **Samples** |
| GSE78213 | CRPC | C4-2 |
| GSE69896 | CRPC | MR49F |
| GSE147876 | CRPC | MR42D |
| GSE162564 | CRPC | 22RV-1 |
| GSE126779 | CRPC | DU145, PC3 |
| Breast cancer | | |
| GEO accession | **Cancer type** | **Samples** |
| GSE171908 | BC | MCF-7, MDA-MB-231 |
| GSE102407 | TNBC | MDA-MB-231 |
| GSE219191 | TNBC | MDA-MB-231 |
| GSE63582 | TNBC | SUM149, SUM159 (parental) |
| GSE123285 | Lum A | MCF-7 |
| GSE235201 | TNBC | HCC70, MDA-MB-231 |
| GSE236250 | TNBC | MDA-MB-231 |
| GSE115550 | TNBC | MDA-MB-231 |
| GSE116907 | TNBC | HCC1806, MDA-MB-231, SUM159 |
| GSE109570 | Lum A | MCF-7 |
| GSE55922 | Lum A | MCF-7 |
| GSE114937 | BC | MDA-MB-231, MDA-MB-453, SKBR3 |
| Prostate cancer | | |
| GEO accession | **Cancer type** | **Samples** |
| GSE78213 | CRPC | C4-2 |
| GSE69896 | CRPC | MR49F |
| GSE147876 | CRPC | MR42D |
| GSE162564 | CRPC | 22RV-1 |
| GSE126779 | CRPC | DU145, PC3 |
| Brain cancer | | |
| GEO accession | **Cancer type** | **Samples** |
| GSE99175 | GBM | U-87 MG |
| GSE245006 | NB | SK-N-BE(2) |
| GSE138942 | GB | U3009MG, U3024MG, U3054MG, U3056MG |
| GSE185024 | MB | D425 |
| GSE107707 | NB | SK-N-BE(2)-C, Kelly (parental) |
| GSE183587 | NB | IMR-32 |
| Skin cancer |  |  |
| GEO accession | **Cancer type** | **Samples** |
| GSE95153 | MEL | M93-047 |
| GSE94488 | MEL | SKmel2, 501MEL, SKmel147 |
| GSE131967 | HNSCC | FaDu, SCC1, SCC22B |
| Gastrointestinal cancer | | |
| GEO accession | **Cancer type** | **Samples** |
| GSE95513 | CRC | RKO |
| GSE118548 | CRC | T84 |
| GSE192903 | PDAC | PANC-1 |
| GSE113215 | GIST | GIST-T1 |

| Gastrointestinal cancer (Continue) | | |
| --- | --- | --- |
| GEO accession | **Cancer type** | **Samples** |
| GSE184065 | HCC | Huh7 |
| GSE158552 | HCC | HepG2 |
| GSE73318 | CRC | COLO205, COLO320, HCT116, HCT-15, HT-29, SW480 |
| GSE255300 | CRC | HCT116, HPCEC, HT29 |
| Lung cancer |  |  |
| GEO accession | **Cancer type** | **Samples** |
| GSE210101 | SCLC | H446 |
| GSE182821 | SCLC | NCI-H1688 |
| GSE197426 | SCLC | NCI-H526 |
| GSE113711 | NSCLC | H23 |
| Lung cancer |  |  |
| GEO accession | **Cancer type** | **Samples** |
| GSE145469 | RMS | Rh30 |
| GSE196057 | AT/RT | BT12 |
| GSE113604 | ES | Cado-ES, CHLA10, RD-ES, SK-N-MC |
| Gynecologic cancer | | |
| GEO accession | **Cancer type** | **Samples** |
| GSE82329 | OC | A1847, A2780, OVCAR5 |
| GSE235031 | CC | HeLa |
| Hematologic cancer | | |
| GEO accession | **Cancer type** | **Samples** |
| GSE188437 | MM | OPM2 |
| GSE79253 | T-ALL | MOLT4 |
| GSE77295 | CML | K562 |
| GSE119744 | CLL | MEC1, MEC2, OSU-CKK, CII |
| GSE97435 | T-ALL | Jurkat |
| GSE70447 | MM | LP-1 |
| GSE92456 | BL | Raji |
| GSE129449 | AML | AML18 |

**Abbreviations:** CRPC, castration-resistant prostate cancer; TNBC, triple-negative breast cancer; Lum A, luminal A breast cancer; BC, breast cancer; GBM, glioblastoma; NB, neuroblastoma; MEL, melanoma; HNSCC, head and neck squamous cell carcinoma; CRC, colorectal cancer; PDAC, pancreatic ductal adenocarcinoma; GIST, gastrointestinal stromal tumor; HCC, hepatocellular carcinoma; SCLC, small cell lung cancer; NSCLC, non-small cell lung cancer; RMS, rhabdomyosarcoma; AT/RT, atypical teratoid/rhabdoid tumor; ES, Ewing sarcoma; OC, ovarian cancer; CC, cervical cancer; MM, multiple myeloma; T-ALL, T-cell acute lymphoblastic leukemia; CML, chronic myeloid leukemia; BL, Burkitt lymphoma; AML, acute myeloid leukemia.

**Table S7.** Publicly available GEO RNA-seq datasets used to analyze *BRD2* expression under BRD4 knockdown and BET inhibition

| GEO accession | Cancer type | Samples |
| --- | --- | --- |
| GSE215393 | RMS | RD |
| GSE50491 | HEK | HEK293T |
| GSE73317 | CRC | HCT116, HT-29 |
| GSE206667 | BC | MCF-7 |
| GSE90444 | PDAC | KP-4 |
| GSE113604 | ES | Cado-ES, CHLA10, RD-ES, SK-N-MC |

**Abbreviations:** RMS, rhabdomyosarcoma; HEK, human embryonic kidney; CRC, colorectal cancer; BC, breast cancer; PDAC, pancreatic ductal adenocarcinoma; ES, Ewing sarcoma

**Table S8.** Publicly available BRD2 and BRD4 ChIP-seq dataset used in this study

| GEO accession | Cancer type | Samples |
| --- | --- | --- |
| GSE131097 | TNBC | SUM149 |

**Abbreviations:** TNBC, triple-negative breast cancer

**Table S9.** ChIP-seq datasets for NFYA, H3K4me3, RNA Pol II occupancy at the *BRD2* locus

| GEO accession | Cancer type | Samples |
| --- | --- | --- |
| GSE31477 (GSM935433) | CML | K562 |
| GSE31477 (GSM935508) | CC | HeLa-S3 |
| GSE31477 (GSM935506) | Lymphoblastoid cells | GM12878 |
| GSE51142 | CRC | LoVo |
| GSE147785 (GSM4445878) | Human embryonic kidney cells | HEK293T |
| GSE152062 (GSM4602027) | Human embryonic kidney cells | HEK293T |

**Abbreviations:** CML, chronic myeloid leukemia; CC, cervial cancer; CRC, colorectal cancer
